# Associations of maternal urinary arsenic concentrations during pregnancy with childhood cognitive abilities: The HOME study

**DOI:** 10.1016/j.ijheh.2022.114009

**Published:** 2022-08-07

**Authors:** Antonio J. Signes-Pastor, Megan E. Romano, Brian Jackson, Joseph M. Braun, Kimberly Yolton, Aimin Chen, Bruce Lanphear, Margaret R. Karagas

**Affiliations:** a Department of Epidemiology, Geisel School of Medicine, Dartmouth College, NH, USA; b Unidad de Epidemiología de la Nutrición. Universidad Miguel Hernández, Alicante, Spain; c CIBER de Epidemiología y Salud Pública (CIBERESP), Instituto de Salud Carlos III (ISCIII), Madrid, Spain; d Instituto de Investigación Sanitaria y Biomédica de Alicante (ISABIAL), Spain; e Department of Earth Sciences, Dartmouth College, Hanover, NH, USA; f Department of Epidemiology, Brown University, Providence, RI, USA; g Department of Pediatrics, Cincinnati Children’s Hospital Medical Center, University of Cincinnati College of Medicine, Cincinnati, OH, USA; h Department of Biostatistics, Epidemiology and Informatics, University of Pennsylvania Perelman School of Medicine, Philadelphia, PA, USA; i Child and Family Research Institute, BC Children’s and Women’s Hospital, Vancouver, BC, Canada; j Faculty of Health Sciences, Simon Fraser University, Burnaby, BC, Canada

**Keywords:** Arsenic, Neurodevelopment, Cognitive abilities, *In utero* exposure, Children

## Abstract

Arsenic exposure during pregnancy may increase the risk for intellectual deficits in children, but limited data exist from prospective epidemiologic studies, particularly at low arsenic exposure levels. We investigated the association between prenatal maternal urinary arsenic concentrations and childhood cognitive abilities in the Health Outcomes and Measures of the Environment (HOME) Study. We used anion exchange chromatography coupled with inductively coupled plasma mass spectrometry detection to measure arsenic species content in pregnant women’s urine. The summation of inorganic arsenic (iAs), monomethylarsonic acid (MMA), and dimethylarsinic acid (DMA) refers to ∑As. We assessed children’s cognitive function (*n* = 260) longitudinally at 1-, 2-, and 3-years using Bayley Scales of Infant and Toddler Development, at 5 years using Wechsler Preschool and Primary Scale of Intelligence, and at 8 years using Wechsler Intelligence Scale for Children. We observed a modest decrease in mental development index and full-scale intelligence quotient at ages 3 and 5 years with each doubling of ∑As with estimated score (ß) differences and 95% confidence interval (CI) of −1.8 from −4.1 to 0.5 and −2.5 from −5.1 to 0.0, respectively. This trend was stronger and reached statistical significance among children whose mothers had lower iAs methylation capacity and low urinary arsenobetaine concentrations. Our findings suggest that arsenic exposure levels relevant to the general US population may affect children’s cognitive abilities.

## 1. Introduction

Arsenic, which occurs in organic and inorganic forms, is ubiquitous (WHO, 2001). Inorganic arsenic (iAs) is an established cause of cancer of the lung, skin, and bladder (IARC, 2012). Also, evidence is growing that iAs is a risk factor for non-cancer health outcomes, such as diabetes and cardiovascular disease (IARC, 2012; Kapaj et al., 2006; Nachman et al., 2017; Ng et al., 2003; Sanchez et al., 2016; Tolins et al., 2014; Tsuji et al., 2015). Arsenic crosses the placenta and enters the fetus (Davis et al., 2014; Gilbert-Diamond et al., 2016; Gluckman et al., 2008; Hall et al., 2007; Punshon et al., 2015; Steinmaus et al., 2014; Vahter, 2008, 2009). Arsenic exposure during early brain development may result in impaired cognitive abilities that last throughout the life course (EFSA, 2009; Freire et al., 2018; Gluckman et al., 2008; Grandjean and Landrigan, 2014; Nachman et al., 2017; Signes-Pastor et al., 2017b; Tolins et al., 2014; Tsuji et al., 2015; Wasserman et al., 2014).

Several countries have established a maximum contaminant level (MCL) of 10 μg/L for arsenic in drinking water. Yet, several million people worldwide consume water with arsenic content above this MCL (Ayotte et al., 2017; EPA, 2001; US EPA, 2012; WHO, 2017, 2011). When arsenic exposure from water and occupation is low, diet becomes the major source (EFSA, 2009; Nachman et al., 2018). Food contains iAs along with several organic forms with variable toxic effects (Cubadda et al., 2016). A multistep process via the one-carbon cycle metabolizes the iAs in the liver. The metabolism cycle generates monomethylarsonic acid (MMA) and dimethylarsinic acid (DMA). Then, the human body excretes them in the urine within a few days along with unmetabolized iAs (Antonelli et al., 2014; Challenger, 1951; Jansen et al., 2016; Tseng, 2009). Hence, urinary arsenic concentration is a widely used biomarker of iAs exposure (Signes-Pastor et al., 2017b, 2017c) and the concentrations ratio of $\frac{\mathrm{MMA}}{\mathrm{iAs}}$ and $\frac{\mathrm{DMA}}{\mathrm{MMA}}$ reflects iAs methylation capacity (Niedzwiecki et al., 2014). The methylation capacity is considered the major iAs detoxification process (Niedzwiecki et al., 2014), and is regulated by the polymorphisms in *AS3MT* gene (Agusa et al., 2011; Jiang et al., 2018; López-Carrillo et al., 2014).

Previous prospective studies on arsenic exposure and childhood neurodevelopment include populations from Bangladesh (Hamadani et al., 2010, 2011; Rodrigues et al., 2016; Tofail et al., 2009; Vahter et al., 2020; Valeri et al., 2017; Wasserman et al., 2016), China (Liang et al., 2020; Wang et al., 2018), Mexico (Levin-Schwartz et al., 2019), Nepal (Parajuli et al., 2013, 2014, 2015), and Spain (Forns et al., 2014; Freire et al., 2018). Most published studies are from contaminated areas with water arsenic above the MCL and show inconsistent findings (Hamadani et al., 2010, 2011; Nahar et al., 2014a, 2014b; Parvez et al., 2011; Rodrigues et al., 2016; Rosado et al., 2007; Tofail et al., 2009; Vahter et al., 2020; Wasserman et al., 2004, 2007). Evidence regarding the effects of arsenic exposure on childhood neurodevelopment among populations with access to low arsenic drinking water is still scarce (Desai et al., 2018, 2020; Forns et al., 2014; Freire et al., 2018; Kordas et al., 2015; Liang et al., 2020; Signes-Pastor et al., 2019; Wasserman et al., 2014).

We hypothesized that higher prenatal arsenic exposure impairs childhood cognitive function in communities with low-level exposure. We also expect that a decreased iAs methylation capacity would exacerbate the toxic effect. To test our hypothesis, we measured maternal urinary arsenic species concentrations in pregnancy and calculated maternal iAs methylation capacity. Then, we evaluated their association with cognitive abilities in US children enrolled in Health Outcomes and Measures of the Environment, the HOME Study, a prospective birth cohort study.

## 2. Methods

### 2.1. Study participants

The HOME Study enrolled pregnant women from the greater metropolitan area of Cincinnati, Ohio between March 2003 and February 2006. The study was designed to investigate the effects of exposure to environmental toxicants on neurodevelopment and other health endpoints in children. Eligibility criteria for HOME Study mothers were i) being ≥18 years old; ii) living in a house built before 1978; iii) having no history of human immunodeficiency virus infection; and iv) not taking medication for seizures or thyroid disorders. Children completed multiple longitudinal follow-up visits through age 12. The visits included assessment of mental, psychomotor, and cognitive development, physical growth, and health conditions (Braun et al., 2017; Chen et al., 2014). The HOME Study enrolled 389 singleton infants and nine sets of twins (Braun et al., 2017); however, only singletons were included in this study. Among singletons (*n* = 389), 310 had pregnancy urinary arsenic concentrations (excluding 79) and 276 at least one cognitive assessment to age 8 years (excluding 34). We also excluded children with missing values in relevant covariates (*n* = 16). The statistical analysis included 260 children (Fig S1). Mothers gave informed consent before enrollment in the study and at postnatal follow-up visits for their children’s participation. The institutional review board for the Cincinnati Children’s Hospital Medical Center and participating hospitals and clinics approved the HOME Study Protocols (i.e, 2015–6165 and 2015–6170).

### 2.2. Sample preparation and chemical analyses

We collected maternal urine samples at 16- and 26-week gestation; however, samples collected at 16-week gestation were only analyzed for arsenic speciation when the 26-week gestation urine samples had insufficient volume. Among the 310 participants with arsenic data, 298 and 12 had their urinary arsenic speciation measured in samples collected at 26- and 16-week gestation, respectively. The Trace Element Analysis Core (TEA) at Dartmouth College determined urinary arsenic speciation (Jackson, 2015; Signes-Pastor et al., 2020). TEA analyzed the urine samples with an Agilent LC 1260 equipped with a Thermo AS7, 2 × 250 mm column and a Thermo AG7, 2 × 50 mm guard column interfaced with an Agilent 8900 inductively coupled plasma mass spectrometry in oxygen reaction cell mode. Each urine samples batch included blanks and replicate samples of certified reference material. The urinary arsenic species included iAs (arsenite + arsenate), and the organic compounds MMA, DMA, and arsenobetaine (AsB). The average (standard deviation) recoveries for the certified reference material NIST 2669 level I (*n* = 38) were 109% (13), 121% (19), 106% (11), and 111% (32) for AsB, DMA, MMA, and iAs, respectively. The average (standard deviation) recoveries for the NIST 2669 level II (*n* = 34) were 102% (10), 97% (11), and 106% (20) for DMA, MMA, and iAs, respectively. The arsenic species limit of detection (LOD) was 0.5 μg/L for iAs, MMA, and DMA, and 0.1 μg/L for AsB. A kinetic Jaffe reaction measured the urine creatinine content (Lausen, 1972).

### 2.3. Cognitive assessment

Children’s cognitive abilities were assessed at ages 1, 2, 3, 5, and 8 years by HOME Study examiners trained and certified by a developmental psychologist (**KY**). We administered the Bayley Scales of Infant and Toddler Development, 2nd edition (Bayley) Mental Development Index (MDI) at 1, 2, and 3 years of age. Intelligence was evaluated using Wechsler Preschool and Primary Scale of Intelligence, 3rd edition (WPPSI) and Wechsler Intelligence Scale for Children, 4th edition (WISC) Full-Scale Intelligence Quotient (FSIQ) at ages 5 and 8 years, respectively (Bayley, 1993; Wechsler, 2003, 2004). Examiners were blinded to the mother’s urinary arsenic concentrations. The Bayley-MDI, WPPSI-FSIQ, and WISC-FSIQ are commonly used in research studies. They provide reliable and valid measures of cognitive function and are statistically equivalent to a population mean of 100 and a standard deviation of 15 (Jiang et al., 2018; Kordas et al., 2015; Parajuli et al., 2015; Tofail et al., 2009; Wasserman et al., 2011, 2018). Prior publications provide further details (Braun et al., 2017; Chen et al., 2014; Nellis and Gridley, 1994).

### 2.4. Statistical analyses

We calculated summary statistics for each variable: median (range and interquartile range) for continuous variables and relative and absolute frequencies for categorical variables. The $\mathrm{LOD}/\sqrt{2}$ value was imputed for statistical analysis when maternal urinary arsenic species concentrations were <LOD (Hornung and Reed, 1990). Maternal sum of urinary arsenic (∑As) was calculated as the summation of arsenate, arsenite, MMA, and DMA. The iAs refers to the summation of arsenate and arsenite, and the primary and secondary methylation indices ($\mathrm{PMI}=\frac{\mathrm{MMA}}{\mathrm{iAs}}$ and $\mathrm{SMI}=\frac{\mathrm{DMA}}{\mathrm{MMA}}$) were calculated as measures for iAs methylation capacity. Maternal urinary arsenic concentrations were positively skewed; thus, they were log_2_-transformed to reduce the influence of extreme values in regression analyses. The MDI and FSIQ scores were normally distributed, and thus transformation was unnecessary.

The dose-response association between arsenic exposure and child cognitive function was evaluated using log_2_-transformed maternal prenatal arsenic concentrations using generalized additive models (GAM) and using tertiles in regression analysis. We observed no strong evidence of non-linearity. Thus, we used linear mixed models to create the regression estimates of maternal urinary ∑As and methylation indices in pregnancy with children’s cognitive function, using unstructured covariance to account for correlation across repeated measurements in the same child. To investigate the association between arsenic exposure and cognitive function at different ages, we included interaction terms between arsenic (continuous) and child age (categorical) in the models. The ∑As, iAs, PMI and SMI were investigated as independent variables in separate regression models. The regression analyses were also performed for each cognitive ability assessment approach individually (i.e., MDI at 1, 2 and 3 years, and FSIQ at 5 and 8 years, respectively).

We identified covariates based on *a priori* associations with exposures and outcomes observed in the literature, previous work investigating neurodevelopmental outcomes in the HOME Study, and the Directed Acyclic Graph using the DAGitty software (Fig. S2) (Desai et al., 2020; Kordas et al., 2015; Liang et al., 2020; Parajuli et al., 2015; Signes-Pastor et al., 2019; Textor et al., 2017; Vahter et al., 2020; Valeri et al., 2017; Wang et al., 2018; Wasserman et al., 2018). We adjusted the models for household income (categorical), maternal race (categorical), maternal age at delivery (continuous), maternal Intelligence Quotient (IQ) measured by Wechsler Abbreviated Scale of Intelligence (continuous), maternal pre-pregnancy body mass index (continuous), log_10_-average serum cotinine in pregnancy based on two time point measurements as an indicator of tobacco smoke exposure, log_10_-urinary creatinine (continuous), Home Observation for Measurement of the Environment score at 1 year - HOME score (continuous), and child sex (binary). Further details regarding covariates can be found in our prior publication (Braun et al., 2017). Models for PMI and SMI were further adjusted for maternal ∑As to account for the overall iAs exposure. Urinary AsB comes from direct ingestion of fish/seafood and does not pose a health risk; however, it is prone to iAs exposure misclassification when urinary arsenic speciation is not performed and total arsenic is used to measure the exposure (Jones et al., 2016; Navas-Acien et al., 2011; Signes-Pastor et al., 2017b, 2019). Here maternal urinary arsenic species concentrations were measured and ∑As excluding AsB was applied to estimate iAs exposure. Fish/seafood may also contain other complex organosenical compounds that are excreted as MMA and DMA after ingestion, thus we performed statistical models restricted to participants with urinary AsB concentrations <1 μg/L suggesting little, or no fish/seafood consumption (Navas-Acien et al., 2011; Signes-Pastor et al., 2020). In sensitivity analysis, we examined maternal blood lead concentration from 16 weeks of gestation as a potential confounder. We also explored the potential effect measure modification of the arsenic-MDI/FSIQ relations by child sex, maternal smoking (i.e., maternal serum cotinine ≥3 ng/mL indicating active smoker status (Benowitz et al., 2008)), and maternal whole blood folate (above/below median of 510 nmol/L). Associations with a nominal level of 0.05 was defined as statistically significant. All statistical analyses were conducted using SAS version 9.4 (SAS Institute Inc., Cary, NC, USA).

## 3. Results

The biochemical, socioeconomic, and anthropometric characteristics of participants included in the analysis (*n* = 260) did not differ from those who were excluded (*n* = 129) (Table S1). Most mothers were non-Hispanic white; 67% of them were within the range of 25–34 years of age. Over 80% of participants’ household income was ≥$20,000/year and were not exposed to tobacco smoke based on serum cotinine levels during pregnancy. Only 9% of women had serum cotinine levels >3 ng/mL, indicative of active tobacco smoking (Benowitz et al., 2008; Braun et al., 2017). Among these women we observed an average (standard deviation) pregnancy serum cotinine concentration of 78 (86) ng/mL, whereas the remaining women (91%) had an average concentration of 0.11 (0.27) ng/mL.

The studied children included 46% males and 54% females. Maternal urinary ∑As had a median (interquartile range) of 3.63 (2.40–5.86) μg/L (Table 1). Maternal urinary MMA concentrations were <0.5 μg/L for almost all participants. Concentrations of urinary arsenic in the HOME Study participants were lower than that noted for women of 18–45 years from NHANES 2003-04 or 2005-06 cycles (Table 2) (NHANES, 2022). The average (standard deviation) scores for MDI and FSIQ were 94 (1), 89 (14), 94 (13), and 103 (15) and 103 (16) at 1, 2, 3, 5 and 8 years of age, respectively.

**Fig. 1.**
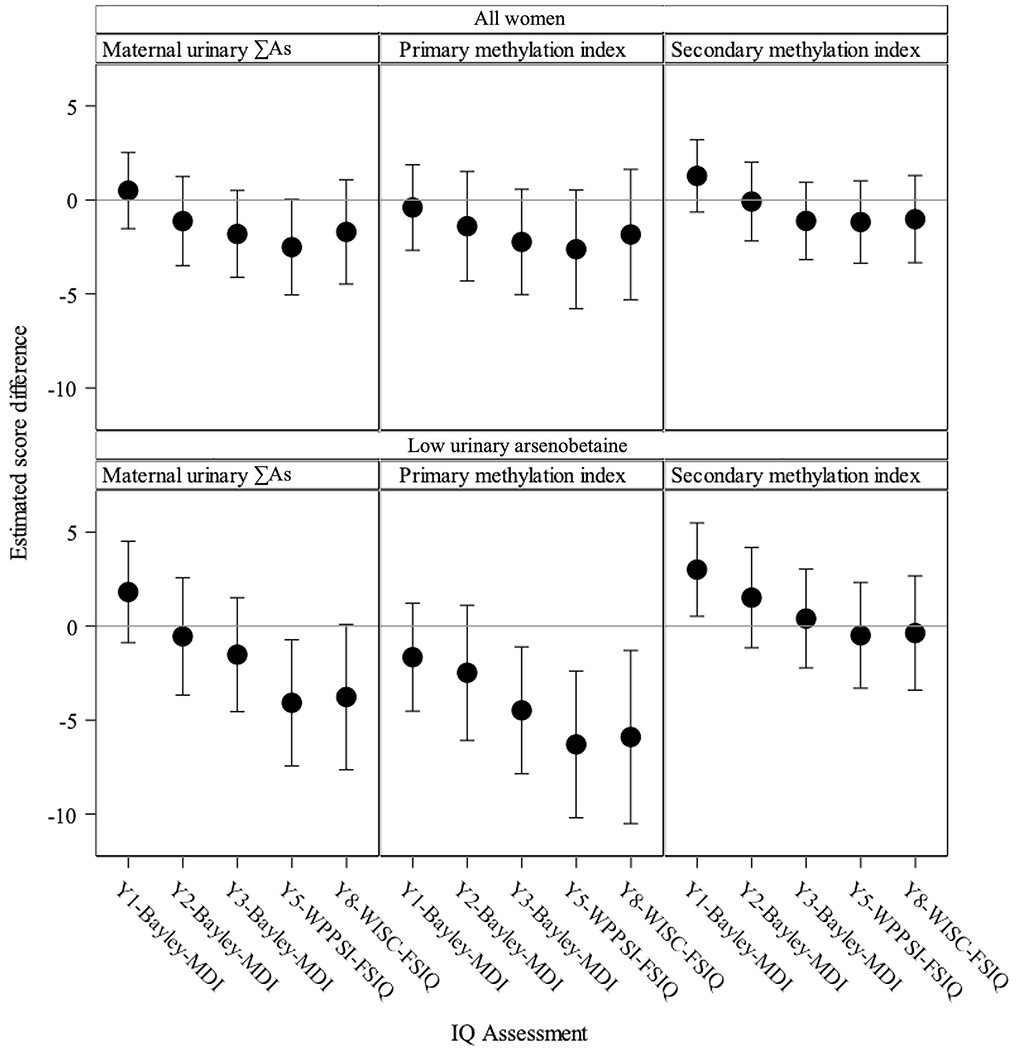
Estimated beta coefficients and 95% CIs for child cognitive scores by a doubling increase in maternal prenatal arsenic concentrations (∑As), HOME Study among all women (*n* = 260) and among women with urinary arsenobetaine concentration <1 μg/L suggesting little, or no fish/seafood consumption (*n* = 167). All estimates are adjusted for household income, maternal race, maternal age at delivery, maternal intelligence quotient measured by Wechsler Abbreviated Scale of Intelligence, maternal pre-pregnancy body mass index (kg/m^2^), log_10_-average serum cotinine in pregnancy (smoking), log_10_-urinary creatinine, HOME score, and child sex. Models for primary and secondary methylation indices are further adjusted for sum of maternal urinary arsenic concentrations (∑As).

A modest, non-statistically significant, decrease in MDI and FSIQ was observed at ages 3 and 5 years with each doubling of ∑As with −1.8 points lower child MDI score (95% confidence interval (CI): −4.1, 0.5) and −2.5 points lower IQ score (95% CI: −5.1, 0.0), respectively (Fig. 1; Table S2). Stronger score reductions, but still not statistically significant, were observed for PMI with −2.2 points lower MDI (95% CI: −5.0, 0.6) and −2.6 points lower FSIQ (95% CI: −5.8, 0.5) compared to SMI with −1.1 points lower MDI (95% CI: −3.2, 0.9) and −1.2 points lower FSIQ (95% CI: −3.4, 1.0) assessed at children’s 3 and 5 year of age, respectively (Fig. 1; Table S2). The estimates from the regression analyses for each cognitive ability assessment approach or timing had a similar pattern of results, though confidence intervals tended to be less precise, likely due to reduced statistical power in these analyses with smaller sample sizes (Table S3).

The overall pattern of results was also consistent among participants with maternal urinary AsB <1 μg/L (*n* = 167). The association of ∑As with MDI at 3 years was attenuated (ß = −1.5; 95% CI: −4.5, 1.5), whereas a doubling of ∑As was associated with a −4.1-point decrease in FSIQ score at 5 years (95% CI: −7.4, −0.7). Statistically significant decreases were observed in children’s MDI at 3 years and FSIQ at 5 and 8 years with each doubling of PMI, with reductions of −4.5 points (95% CI: −7.9, −1.1), −6.3 points (95% CI: −10.2, −2.4), and −5.9 points (95% CI: −10.5, −1.3), respectively (Fig. 1; Table S2). However, differences were not observed with SMI (Fig. 1; Table S2).

In our population, we observed average (standard deviation) maternal blood lead of 0.69 (0.31) μg/dL, and our sensitivity analyses showed that blood lead was weakly correlated with urinary ∑As (r = 0.13, *p*-value = 0.10), but did not correlate with urinary iAs, PMI or SMI (r < 0.08, *p*-value >0.18). The inclusion of maternal blood lead in the multivariable models did not change the regression coefficients for associations of any arsenic measure with MDI/FSIQ by >10% (Fig. S3). The analysis did not show evidence of effect measure modification of the associations of interest by child sex, maternal smoking, or maternal whole blood folate (data not shown).

## 4. Discussions

Fetal exposure to environmental toxicants such as arsenic may impact brain development with a marked effect throughout the lifespan (Grandjean and Landrigan, 2006, 2014). Oxidative stress, apoptosis, thiamine deficiency, and decreased acetyl cholinesterase activity are suggested arsenic-induced neurotoxic mechanisms (Ahmed et al., 2011; Mochizuki, 2019; Singh et al., 2011). Prior studies suggest that arsenic exposure associates with impaired cognitive abilities in populations living in water arsenic-contaminated regions (Hamadani et al., 2011; Nahar et al., 2014a, 2014b; Parvez et al., 2011; Rodrigues et al., 2016; Rosado et al., 2007; Vahter et al., 2020; Wasserman et al., 2004, 2007). However, the effects of arsenic neurotoxicity during vulnerable windows at levels relevant to the general US population and others, where public water arsenic concentrations are below 10 μg/L (EPA, 2001; US EPA, 2012; WHO, 2017, 2011), are not well established (Desai et al., 2018, 2020; Forns et al., 2014; Freire et al., 2018; Kordas et al., 2015; Liang et al., 2020; Signes-Pastor et al., 2019; Wasserman et al., 2014). While maternal urinary arsenic concentrations during pregnancy were relatively low in our study, they related to reduced cognitive scores during childhood. There was evidence that a lower maternal iAs methylation capacity may exacerbate the adverse effects.

In the present study, we did not observe a clear association between gestational arsenic exposure at levels relevant to the general US population and children’s MDI at 1 and 2 years of age, but pregnancy urinary arsenic concentrations were associated with a reduction in MDI at 3 years, and FSIQ at 5 and 8 years of age. Other studies also reported that children ≥3 years of age showed impaired cognitive abilities related to prenatal exposure to toxicants such as mercury, polybrominated diphenyl ether (PBDEs), and chlorpyrifos, but not at earlier ages (Chen et al., 2014; Karagas et al., 2012; Rauh et al., 2006). While we were not able to consider these factors in our analysis, we do not anticipate they would be strongly associated with arsenic concentrations.

Although we did not observe associations between maternal urinary arsenic concentrations and cognitive abilities until age 3, some prior work from China (Liang et al., 2020; Wang et al., 2018), Nepal (Parajuli et al., 2013), and Bangladesh (Rodrigues et al., 2016; Valeri et al., 2017) found that gestational arsenic exposure at various levels may have an impact at earlier time points. In mother-infant pairs, cord blood arsenic concentrations related to a decrease in neonatal neurobehavioral scores (Wang et al., 2018) and increased risk of personal-social function at 6 months of age in China (Liang et al., 2020). Cord blood arsenic also related to reduced behavior responses and reflex scores at birth in Nepal (Parajuli et al., 2013), but the latter did not persist at 6 or 36 months of age (Parajuli et al., 2014, 2015). Studies from Bangladesh reported reduced IQ scores in 5-year-old children associated with urinary arsenic during pregnancy (Hamadani et al., 2011), but no relation with mental and psychomotor development indices at 18 months of age (Hamadani et al., 2010). Also, from Bangladesh, drinking water arsenic during pregnancy and cord blood and urine concentrations related to reduced cognitive function in children of ~3 (Rodrigues et al., 2016; Valeri et al., 2017) and ~10 (Vahter et al., 2020) years of age. However, another study from Bangladesh did not detect effects of gestational arsenic exposure assessed with maternal urinary arsenic on infants’ problem-solving ability and motor development at 7 months (Tofail et al., 2009). Differences across neurodevelopmental domains, biological matrices used for exposure assessment, exposure levels, or participant characteristic across studies could in part explain these inconsistencies.

Among populations with lower levels of exposure, a study from Spain observed that detectable placenta arsenic concentrations were associated with impaired global and verbal executive abilities in children of 4-5-years of age (Freire et al., 2018). However, a prior study did not observe clear associations with maternal total urinary arsenic, which included AsB, and raises concerns of iAs exposure misclassification in this study (Forns et al., 2014). In the present study, we analyzed urinary arsenic species concentrations and calculated the summation of urinary iAs metabolites (i.e., iAs, MMA, and DMA excluding AsB) as a proxy for iAs exposure. In addition, we performed analysis restricted to women who were low consumers of fish/seafood (AsB <1 μg/L) (Navas-Acien et al., 2011; Signes-Pastor et al., 2020). In the above analysis, we observed stronger inverse associations of ∑As with FSIQ at 5 years and of PMI with MDI at 3 years and FSIQ at 5 and 8 years. Although, this sensitivity analysis was likely underpowered given the reduction in sample size, it suggests that accounting for the association of fish/seafood consumption with arsenic exposure and neurodevelopment may be critically important for future research studies, especially among populations whose diets play a major role in arsenic exposure.

In this study, we found that a diminished iAs methylation capacity in mothers was inversely associated with child cognitive abilities. In humans, there is large inter-individual variation in methylation capacity of iAs and is characterized by the formation of DMA (60–70%) and MMA (10–20%) excreted along with unmetabolized iAs (10–30%) (Signes-Pastor et al., 2017a; Vahter, 2002). Altered profiles of urinary arsenic species in urine, which are genetically driven, appear to reflect differences in the efficacy of iAs metabolism (Agusa et al., 2011). In Taiwan, a stronger methylation capacity defined as higher urinary DMA% in 2-year-old children related to an increased cognitive and fine motor (Jiang et al., 2018). Thus, it is necessary to consider iAs methylation capacity when investigating the neurotoxicity of arsenic.

We did not have data on childhood exposure. However, prior studies suggest an inverse association between arsenic exposure during childhood and impaired neurodevelopment. Among ≤5-year-old children, urinary arsenic (median of 4.85 μg/L) related to a decreased in motor functions in Spain (Signes-Pastor et al., 2019). Urinary arsenic concentrations among 7-year-old children (median of 9.9 μg/L) were inversely associated with executive function in Uruguay (Desai et al., 2020), but not with the cognition (Desai et al., 2018; Kordas et al., 2015) in accordance with a recent study from China (Zhou et al., 2020). Reduced IQ and behavior scores were reported to be associated with children’s biomarkers of arsenic exposure (e.g., blood, urine, nails, and hair) in Bangladesh (Hamadani et al., 2011; Nahar et al., 2014a, 2014b; Nahar and Inaoka, 2012; Vahter et al., 2020; Wasserman et al., 2011, 2016, 2018), India (Ghosh et al., 2017; Manju et al., 2017) and Mexico (Calderón et al., 2001; Roy et al., 2011). In the US, children consuming water arsenic ≥5 μg/L had lower IQ scores compared to those consuming water arsenic <5 μg/L (Wasserman et al., 2014). Several studies from China (Wang et al., 2007), India (Ehrenstein et al., 2007), Taiwan (Tsai et al., 2003), Bangladesh (Wasserman et al., 2004, 2007), and Mexico (Rocha-Amador et al., 2007) reported impaired cognitive ability associated with water arsenic exposure. A recent dose-response meta-analysis described a 0.08% decrease in IQ scale associated with each 1 μg/L increase in water arsenic concentration (Hasanvand et al., 2020). Studies from Italy (Lucchini et al., 2019) and Mexico (Rosado et al., 2007; Roy et al., 2011) found that proximity to industrial arsenic emissions may also affect children’s cognitive abilities.

Exposure to environmental toxicants occur simultaneously as a mixture in real-life scenarios and their health impact may relate to the concentrations of each component of the mixture (Levin-Schwartz et al., 2019; Valeri et al., 2017; Wasserman et al., 2018). A negative effect of a mixture of arsenic, lead, and manganese assessed using cord blood concentrations, on children’s cognitive abilities was reported in a Bangladesh study (Valeri et al., 2017), and an additional study suggested that arsenic and cadmium exposures are the most important mixture components associated with a decrease in adolescent intelligence when applying the same flexible statistical methods (Wasserman et al., 2018). Other studies have applied multivariable-adjusted regression models to account for multiple exposures (Freire et al., 2018; Parajuli et al., 2015; Vahter et al., 2020). While little is known about the impact of multiple metal exposure, including arsenic, at relatively low levels on the development of cognitive abilities in childhood, in our study, maternal blood Pb concentrations did not appear to influence observed associations of arsenic with childhood cognition, but other neurotoxicants could confound or modify the effect of arsenic.

This study is based on a well-characterized US cohort (Braun et al., 2017) that counted on extensively trained research personnel to longitudinally assess children’s cognitive abilities using established quality assurance/quality control (QA/QC) protocols (Bayley, 1993; Braun et al., 2017; Wechsler, 2003, 2004), and measured urinary arsenic species concentrations. While our findings are based on a modest sample size, we nevertheless observed that gestational exposure to arsenic may impair children’s cognitive abilities, especially among older children whose mother had lower methylation capacity when adjusting for several potential confounding factors. Still, the effect of unknown factors or residual confounding, including from unknown or unmeasured co-exposures, remains a possibility. Our findings are among the first to suggest that even low-level arsenic exposure during vulnerable windows of growth and development may adversely impact children’s congnitive abilities (Desai et al., 2020; Freire et al., 2018; Signes-Pastor et al., 2019; Wasserman et al., 2014). More prospective research is needed to confirm the relevant windows of exposure from gestation to early life on arsenic neurotoxicity at levels relevant to the general population and to evaluate cumulative exposures and mixture effects.

## Supplementary Material

Supplementary information

## Figures and Tables

**Table 1:** Maternal urinary arsenic concentrations (∑As) in pregnancy according to maternal and children’s factors, HOME Study.

Characteristics	*n* (%)^a^	∑As (μg/L) Median (IQR)^b^
**All participants**	260 (100)	3.63 (2.40–5.86)
**Maternal age at delivery (years)**		
< 25	47 (18)	4.62 (2.82–6.39)
25-34	173 (67)	3.52 (2.43–5.56)
≥ 35	40 (15)	3.33 (1.78–6.60)
**Maternal race/ethnicity**		
Non-Hispanic white	185 (71)	3.16 (2.23–5.27)
Non-Hispanic black and others	75 (29)	5.17 (3.34–7.22)
**Maternal education**		
High school or less	42 (16)	5.59 (2.93–7.65)
Some college or 2-year degree	62 (24)	3.86 (2.82–5.26)
Bachelor’s	92 (36)	3.18 (2.32–6.40)
Graduate or professional	64 (25)	3.20 (2.14–4.86)
**Maternal marital status**		
Married or living with partner	224 (86)	3.48 (2.32–5.63)
Not married and living alone	36 (14)	5.06 (3.10–6.95)
**Household income**		
< $20,000	41 (16)	5.28 (3.00–7.27)
$20,000–79,999	137 (53)	3.63 (2.54–5.43)
≥ $80,000	82 (32)	3.07 (2.14–5.86)
**Child sex**		
Male	119 (46)	3.74 (2.43–6.39)
Female	141 (54)	3.61 (2.40–5.63)

a At enrollment.

b Sum of iAs (arsenate + arsenite), MMA and DMA.

**Table 2:** Urinary arsenic species concentrations in the HOME Study pregnant women enrolled between March 2003, and February 2006 and in women of 18–45 years of age from NHANES 2003-04 and 2005-06 cycles.

Urinary Arsenic (μg/L)	NHANES 2003-04^a^	NHANES 2005-06^a^	HOME Study				

	*n* = 436	*n* = 532	*n* = 260				
	Median (95% CI)	Median (95% CI)	Median (95% CI)	25th percentile	75th percentile	% <LOD	LOD
∑As ^b^	6.10 (5.7–7.10)	6.18 (5.41–7.17)	3.63 (3.19–4.06)	2.40	5.86	–	–
iAs^c^	1.50 (1.50–2.10)	1.56 (1.56–2.26)	0.87 (0.71–0.92)	0.71	1.06	–	–
DMA	3.80 (3.00–4.00)	3.73 (3.27–4.63)	2.27 (1.94–2.75)	1.13	4.27	8%	0.5
MMA	0.60 (0.60–1.10)	0.64 (0.64–1.10)	<0.5	<0.5	0.53	74%	0.5
AsB	0.90 (0.70–1.40)	2.06 (1.19–2.87)	0.53 (0.35–0.78)	<0.5	2.29	47%	0.5

a NHANES data (NHANES, 2022). The NHANES urinary arsenic concentrations descriptive statistics were calculated using the “survey” package in R version 4.0.3 to account for the sample weights. The NHANES 2003-04 cycle contains 418 (96.87%) arsenite, 407 (93.34%) arsenate, 287 (65.82%) monomethylarsonic acid (MMA), 57 (13.07%) dimethylarsinic acid (DMA), and 138 (31.65%) arsenobetaine (AsB) values below the limit of detection (<LOD). The NHANES 2005-06 cycle contains 520 (97.74%) arsenite, 509 (95.67%) arsenate, 375 (70.48%) MMA, 74 (13.90%) DMA, and 152 (28.57%) AsB values < LOD.

b Sum of iAs, MMA, and DMA.

c Sum of arsenate and arsenite.
