# Supplementary material for: Associations of maternal urinary arsenic concentrations during pregnancy with childhood cognitive abilities: The HOME study
Source: Int J Hyg Environ Health. Author manuscript; Available in PMC 2022 Sep 23. (PMC9500348; doi:10.1016/j.ijheh.2022.114009)
Supplement: Supplementary information [file NIHMS1837049-supplement-Supplementary_information.docx]

# Associations of maternal urinary arsenic concentrations during pregnancy with childhood cognitive abilities: The HOME Study

Antonio J. Signes-Pastor^1, 2†^, Megan E. Romano^1†^, Brian Jackson^3^, Joseph M. Braun^4^, Kimberly Yolton^5^, Aimin Chen^6^, Bruce Lanphear^7^, Margaret R. Karagas^1^

^1^Department of Epidemiology, Geisel School of Medicine, Dartmouth College, NH, USA. ^2^Unidad de Epidemiología de la Nutrición. Universidad Miguel Hernández, Alicante, Spain. CIBER de Epidemiología y Salud Pública (CIBERESP), Instituto de Salud Carlos III (ISCIII), Madrid, Spain. Instituto de Investigación Sanitaria y Biomédica de Alicante (ISABIAL), Spain. ^3^Department of Earth Sciences, Dartmouth College, Hanover, NH, USA. ^4^Department of Epidemiology, Brown University, Providence, RI, USA. ^5^Department of Pediatrics, Cincinnati Children’s Hospital Medical Center, University of Cincinnati College of Medicine, Cincinnati, OH, USA. ^6^Department of Biostatistics, Epidemiology and Informatics, University of Pennsylvania Perelman School of Medicine, Philadelphia, PA, USA. ^7^Child and Family Research Institute, BC Children’s and Women’s Hospital, Vancouver, BC, Canada, and Faculty of Health Sciences, Simon Fraser University, Burnaby, BC, Canada.

^†^Antonio J. Signes-Pastor ([antonio.j.signes-pastor@dartmouth.edu](mailto:antonio.j.signes-pastor@dartmouth.edu) & [asignes@umh.es](mailto:asignes@umh.es)) and Megan E. Romano ([megan.e.romano@dartmouth.edu](mailto:megan.e.romano@dartmouth.edu)) share first authorship.

**Supplemental material**

## Table S1: Maternal urinary arsenic concentrations (∑As) in pregnancy according to maternal sociodemographic and children’s factors overall and excluded participants, HOME Study.

| **Characteristics** | ***n* at enrollment (%)** | **Maternal ∑As (µg/L) Median (IQR)** | ***n* excluded with arsenic data (%)** | **Maternal ∑As (µg/L) Median (IQR)** | **Total *n* excluded (%)** |
| --- | --- | --- | --- | --- | --- |
| **All participants** | 260 (100) | 3.63 (2.40-5.86) | 34 (100) | 4.16 (2.71-5.62) | 129 (100) |
| **Maternal age (years)** |  |  |  |  |  |
| <25 | 47 (18) | 4.62 (2.82-6.39) | 24 (48) | 3.68 (2.70-4.74) | 49 (38) |
| 25-34 | 173 (67) | 3.52 (2.43-5.56) | 20 (40) | 4.67 (2.50-5.73) | 57 (45) |
| ≥35 | 40 (15) | 3.33 (1.78-6.60) | 6 (12) | 4.70 (4.05-6.34) | 21 (16) |
| **Maternal race/ethnicity** |  |  |  |  |  |
| Non-Hispanic white | 185 (71) | 3.16 (2.23-5.27) | 33 (66) | 4.05 (2.87-5.23) | 51 (40) |
| Non-Hispanic black and others | 75 (29) | 5.17 (3.34-7.22) | 17 (34) | 4.40 (2.51-6.34) | 77 (60) |
| **Maternal education** |  |  |  |  |  |
| High school or less | 42 (16) | 5.59 (2.93-7.65) | 29 (58) | 3.33 (2.68-4.96) | 64 (50) |
| Some college or 2-year degree | 62 (24) | 3.86 (2.82-5.26) | 11 (22) | 4.64 (3.88-5.72) | 29 (23) |
| Bachelor’s | 92 (36) | 3.18 (2.32-6.40) | 4 (8) | 3.28 (2.29-7.80) | 18 (14) |
| Graduate or professional | 64 (25) | 3.20 (2.14-4.86) | 6 (12) | 5.96 (3.83-10.17) | 17 (13) |
| **Maternal marital status** |  |  |  |  |  |
| Married or living with partner | 224 (86) | 3.48 (2.32-5.63) | 27 (54) | 4.10 (2.90-5.62) | 79 (62) |
| Not married and living alone | 36 (14) | 5.06 (3.10-6.95) | 19 (38) | 4.38 (3.17-5.74) | 43 (34) |
| **Household income** |  |  |  |  |  |
| <$20,000 | 41 (16) | 5.28 (3.00-7.27) | 16 (32) | 4.34 (2.70-5.49) | 79 (62) |
| $20,000-79,999 | 137 (53) | 3.63 (2.54-5.43) | 23 (46) | 4.05 (2.91-5.62) | 56 (44) |
| ≥$80,000 | 82 (32) | 3.07 (2.14-5.86) | 7 (14) | 5.57 (3.83-10.17) | 21 (16) |
| **Children sex** |  |  |  |  |  |
| Male | 119 (46) | 3.74 (2.43-6.39) | 25 (50) | 4.23 (3.17-5.23) | 61 (48) |
| Female | 141 (54) | 3.61 (2.40-5.63) | 25 (50) | 3.88 (2.51-5.72) | 66 (52) |

## Table S2: Estimated beta coefficients and 95% CIs in child cognitive scores by a doubling increase in maternal arsenic concentrations (∑As) in pregnancy, HOME Study among all women (*n* = 260) and among women with urinary arsenobetaine (AsB) concentration <1 μg/L suggesting little, or no fish/seafood consumption (*n* =167).

| **All Women** |  |  |  |  |
| --- | --- | --- | --- | --- |
| **Assessment and age (years)** | ∑As | iAs | PMI | SMI |
| MDI at age 1 years | 0.5 (-1.5, 2.5) | 0.2 (-2.9, 3.2) | -0.4 (-2.7, 1.9) | 1.3 (-0.6, 3.2) |
| MDI at age 2 years | -1.1 (-3.5, 1.2) | 0.5 (-3.4, 4.4) | -1.4 (-4.3, 1.5) | -0.1 (-2.2, 2.0) |
| MDI at age 3 years | -1.8 (-4.1, 0.5) | 2.7 (-1.1, 6.5) | -2.2 (-5.0, 0.6) | -1.1 (-3.2, 0.9) |
| FSIQ at age 5 years | -2.5 (-5.1, 0.0) | 1.2 (-3.1, 5.6) | -2.6 (-5.8, 0.5) | -1.2 (-3.4, 1.0) |
| FSIQ at age 8 years | -1.7 (-4.5, 1.1) | 2.2 (-2.5, 6.8) | -1.8 (-5.3, 1.6) | -1.0 (-3.3, 1.3) |
| **Women with urinary AsB concentration <1 μg/L** |  |  |  |  |
| Assessment and age (years) |  |  |  |  |
| MDI at age 1 years | 1.8 (-0.9, 4.5) | 2.2 (-2.1, 6.4) | -1.7 (-4.5, 1.2) | 3.0 (0.5, 5.5) |
| MDI at age 2 years | -0.5 (-3.7, 2.6) | 0.7 (-4.9, 6.2) | -2.5 (-6.1, 1.1) | 1.5 (-1.2, 4.2) |
| MDI at age 3 years | -1.5 (-4.5, 1.5) | 4.9 (-0.4, 10.2) | -4.5 (-7.9, -1.1) | 0.4 (-2.2, 3.0) |
| FSIQ at age 5 years | -4.1 (-7.4, -0.7) | 2.9 (-3.4, 9.2) | -6.3 (-10.2, -2.4) | -0.5 (-3.3, 2.3) |
| FSIQ at age 8 years | -3.8 (-7.6, 0.1) | 2.6 (-4.6, 9.8) | -5.9 (-10.5, -1.3) | -0.4 (-3.4, 2.7) |

All estimates are adjusted for household income, maternal race, maternal age at delivery, maternal intelligence quotient measured by Wechsler Abbreviated Scale of Intelligence, maternal pre-pregnancy body mass index (kg/m^2^), log_10_-average serum cotinine in pregnancy (smoking), log_10_-urinary creatinine, HOME score, and child sex. Models for primary and secondary methylation indices are further adjusted for sum of maternal urinary arsenic concentrations (∑As).

## Table S3: Estimated beta coefficients and 95% CIs in child cognitive scores for each assessment method by a doubling increase in maternal arsenic concentrations (∑As) in pregnancy.

| Assessment and age (years) | ∑As | iAs | PMI | SMI |
| --- | --- | --- | --- | --- |
| MDI |  |  |  |  |
| at age 1 years | -0.6 (-2.4, 1.2) | 0.2 (-2.8, 3.2) | -0.5 (-2.8, 1.7) | 1.3 (-0.6, 3.3) |
| at age 2 years | -1.3 (-3.7, 1.1) | 0.2 (-3.8, 4.1) | -1.4 (-4.4, 1.5) | 0.1 (-2.1, 2.2) |
| at age 3 years | -2.0 (-4.3, 0.3) | 2.3 (-1.6, 6.1) | -2.3 (-5.1, 0.6) | -1.0 (-3.1, 1.1) |
| FSIQ |  |  |  |  |
| at age 5 years | -1.4 (-4.3, 1.5) | 0.4 (-3.9, 4.7) | -1.5 (-4.6, 1.7) | 0.0 (-2.9, 2.9) |
| at age 8 years | -0.6 (-3.6, 2.5) | 1.6 (-3.0, 6.2) | -0.7 (-4.1, 2.7) | 0.1 (-2.9, 3.1) |

Individual estimated beta coefficients and 95% CIs for child MDI (*n* = 257) and FSIQ (*n* = 194) scores by a 2-fold increase in maternal prenatal arsenic concentrations. Notice that the sample sizes are driven by the follow-up data availability. All estimates are adjusted for household income, maternal race, maternal age at delivery, maternal intelligence quotient measured by Wechsler Abbreviated Scale of Intelligence, maternal pre-pregnancy body mass index (kg/m^2^), log_10_-average serum cotinine in pregnancy (smoking), log_10_-urinary creatinine, HOME score, and child sex. Models for primary and secondary methylation indices are further adjusted for maternal total urinary arsenic concentration.

## Figure S1: Flowchart participants


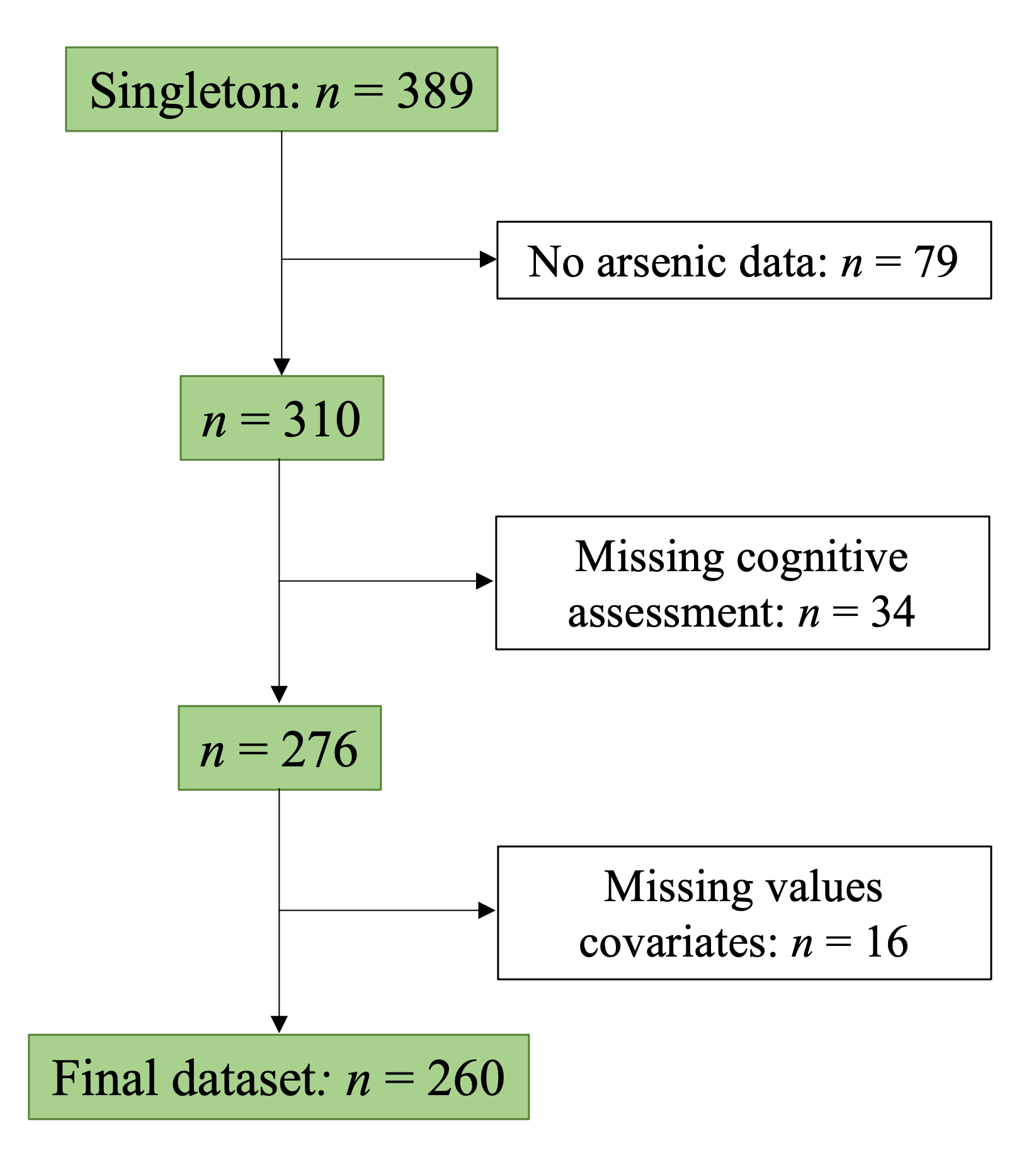


## Figure S2: Directed acyclic graph or causal Bayesian network created using DAGitty browser-based environment.


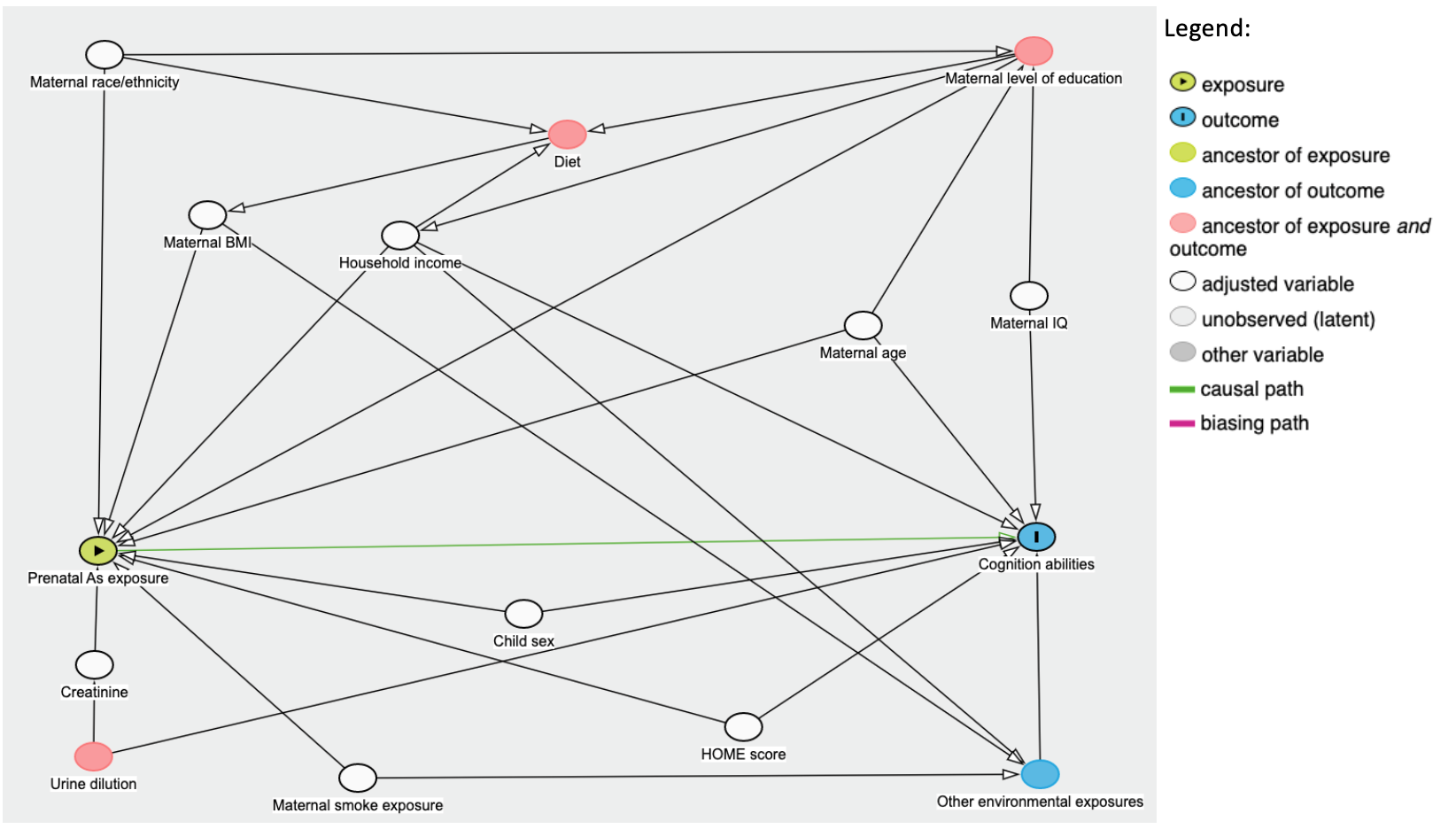


## Figure S3: Estimated beta coefficients and 95% CIs for child cognitive scores by a doubling increase in maternal arsenic concentrations (∑As) in pregnancy adjusted for maternal blood lead concentration, HOME Study (*n* = 260).


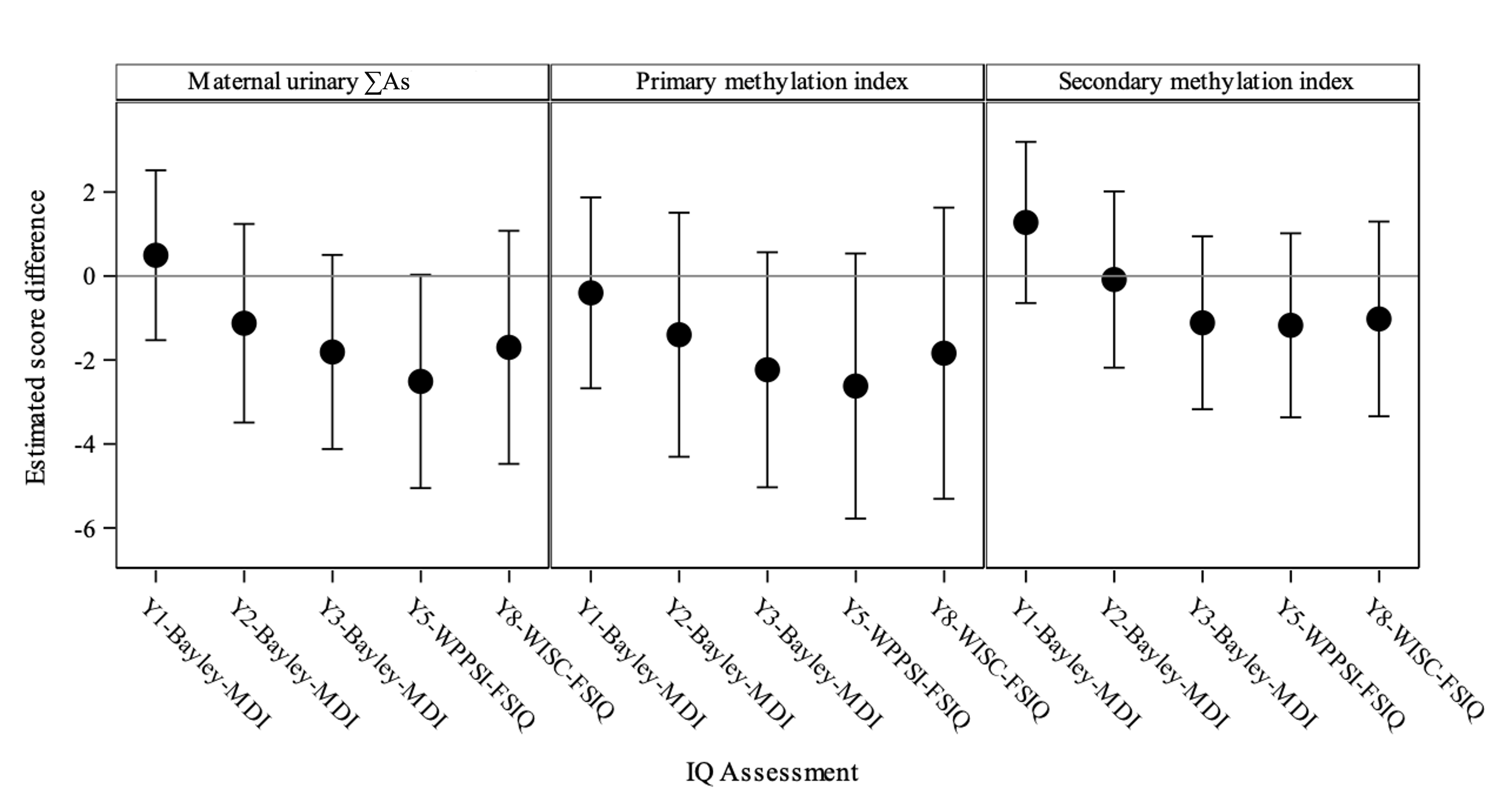


All estimates are adjusted for maternal blood lead in pregnancy, household income, maternal race, maternal age at delivery, maternal intelligence quotient measured by Wechsler Abbreviated Scale of Intelligence, maternal pre-pregnancy body mass index (kg/m^2^), log_10_-average serum cotinine in pregnancy (smoking), log_10_-urinary creatinine, HOME score, and child sex. Models for primary and secondary methylation indices are further adjusted for maternal urinary ∑As.
